# Moderating effects of perceived social support on self-efficacy and psychological well-being of Chinese nurses: a cross-sectional study

**DOI:** 10.3389/fpubh.2023.1207723

**Published:** 2023-09-29

**Authors:** Jiachen Lu, Bin Wang, Xiaofeng Dou, Yingying Yu, Yanni Zhang, Haoqiang Ji, Xu Chen, Meng Sun, Yuxin Duan, Yuanping Pan, Yunting Chen, Yaohui Yi, Ling Zhou

**Affiliations:** ^1^School of Public Health, Dalian Medical University, Dalian, China; ^2^Department of Nursing, The First Affiliated Hospital of Dalian Medical University, Dalian, China; ^3^Laboratory Animal Center, Affiliated Zhongshan Hospital Dalian University, Dalian, China; ^4^School of Public Health, Shandong University, Jinan, China

**Keywords:** moderating effect, nurse, perceived social support, psychological well-being, self-efficacy

## Abstract

**Introduction:**

Nurses experience significant physical and psychological stress that negatively influences their psychological well-being. The objective of this study was to explore the association between self-efficacy and psychological well-being among Chinese nurses and to assess the moderating effects of perceived social support (PSS).

**Methods:**

In 2020, a hospital-based cross-sectional study using a multistage random sampling approach was performed in five regions of Liaoning, China. Of the 1,200 surveyed nurses, 1,010 completed questionnaires that evaluated the demographic information, 14-item Hospital Anxiety and Depression Scale, General Self-Efficacy Scale, and Multidimensional Scale of Perceived Social Support. To examine the factors associated with mental health parameters, hierarchical multiple regression analysis was performed. The interactions were visualized using a simple slope analysis.

**Results:**

The mean depression and anxiety scores for Chinese nurses were 8.74 ± 3.50 and 6.18 ± 3.26, respectively. The association between self-efficacy and depression differed between the low perceived social support (PSS) group (1 SD below the mean, *β =* −0.169, *p* < 0.01) and high PSS group (1 SD above the mean, *β =* −0.077, *p* < 0.01). Similarly, the association between self-efficacy and anxiety differed between the low PSS group (1 SD below the mean, *β* = −0.155, *p* < 0.01) and high PSS group (1 SD above the mean, *β* = −0.044, *p* < 0.01).

**Conclusion:**

We found that Chinese nurses experienced high levels of anxiety and depression. Furthermore, PSS moderates the relationship between self-efficacy and psychological well-being. Therefore, interventions targeting self-efficacy and PSS should be implemented to improve the psychological well-being of nurses.

## 1. Introduction

Depression and anxiety are the first and sixth leading causes of nonfatal diseases worldwide, respectively. They are more common in women than in men (1), typically occurring together and affecting various populations. In both developing and developed countries, nurses are under the most intense stress among all professions and are more likely to experience mental health problems than the general population and other healthcare professionals (2). Of all psychological problems, anxiety and depression have the highest incidence and have the most important implications for mental health research in nurses (3).

Epidemiological studies from different countries have shown that 17–65% of nurses have depressive symptoms (4, 5) and 20–70% have anxiety symptoms (6, 7). In China, the prevalence rates of anxiety and depression among nurses are 32.8–43.4 and 38%, respectively (8). The impact of anxiety and depression on nurses and healthcare organizations has been widely documented. High levels of depression and anxiety are associated with cognitive impairment in specific domains, such as attention and memory (9). In nurses, poor mental health can affect the professional performance and quality of care being provided by them, leading to impaired performance, errors in judgment, and even workplace accidents (5, 10). In addition, the mental health of nurses can jeopardize patient satisfaction and lives, damaging the reputation of healthcare institutions and leading to poor clinical outcomes and productivity. Nurses are also at risk of bullying, which can lead to mental disorders (11, 12). Mental disorders are significantly associated with burnout and high turnover rates, contributing to the long-standing shortage of nurses in many countries, particularly China (13, 14). Nurses serve as the primary point of care for patients, improving the quality of healthcare and fostering a positive doctor-patient relationship (15). However, the hospital work environment is stressful and demanding, with unrealistic expectations and demands from patients and their families. Additionally, caring for emergency and terminally ill patients and witnessing patient deaths and other distressing events can result in unfavorable mental health conditions for nurses who maintain prolonged patient contact (16). Compared to certain developed countries, the relationship between nurses and patients in China may be more strained, and workplace violence against nurses has become a prevalent and significant public health issue (17). Workplace violence contributes to the development of anxiety, depression, and other psychological disorders among nurses in China. Therefore, with the increasing age and healthcare demand of the population, the mental health of nurses requires urgent attention.

There is substantial evidence that shift work (SW), low organizational justice, high job stress, high effort/reward imbalance, and sleep disturbances may increase the risks of anxiety and depression among nurses (18), whereas social capital, resilience, and self-esteem may lead to positive mental health or prevent certain negative outcomes (19). Because anxiety and depression affect the mental and physical health of nurses, it is important to identify positive internal and external resources that counteract these negative effects. Self-efficacy has certain effects on the cognition, emotions, and behavior of nurses, enabling individuals to deal with stressful situations. There is evidence that self-efficacy and anxiety symptoms are negatively correlated. Therefore, low self-efficacy is usually accompanied by high levels of anxiety symptoms (20). Similarly, a multicenter cross-sectional study demonstrated an association between depression and low levels of general self-efficacy and self-evaluation among nurses (21). The general self-efficacy significantly affects the mental health of nurses. High self-efficacy empowers nurses to maintain a stable mood amid stressors and to experience positive emotions. Individuals with high levels of self-efficacy usually perceive painful stimuli as a challenge, promoting the acceptance of the situation, achievement of goals, and reduction in the incidence of anxiety and depression. Social support has positive effects on stress, health (22), sense of belonging and work attitudes of employees, psychological stress, burnout (23, 24), and psychological well-being. It can also protect individuals from threats to their physical and mental health by reducing or offsetting the negative effects of stressful events experienced at work. It has also been shown to protect the mental health of nurses (25). Feng et al. (26) found that the perceived social support (PSS) among medical professionals reduces the impact of psychological distress. Hou et al. (27) demonstrated that PSS has a direct impact on mental health and mediates the indirect effects of resilience on anxiety, partially through PSS. The moderating role of PSS was demonstrated in a study of adolescents, where PSS moderated the relationship between parental scolding and adolescent depression (28). Individuals with high PSS levels were less likely to experience stress-related health problems (29). Thus, PSS moderates the significant association between self-efficacy and anxiety and depression among Chinese nurses.

Overall, the development of self-efficacy is considered a promising approach for enhancing the ability of individuals to cope with anxiety and depression. Previous research has shown that PSS is a critical factor influencing anxiety and depression, which can moderate the association between self-efficacy and mental health. Although previous studies have demonstrated the relationships between general self-efficacy and mental health, as well as between PSS and mental health, the interrelationships of self-efficacy, PSS, and mental health have not been extensively studied among nurses. Establishing that PSS is an external factor and general self-efficacy is an internal psychological source that significantly impacts the psychological state of nurses could provide valuable insight in reducing anxiety and depression among nurses in the current complex and demanding professional environment. Such findings can enhance our understanding of the challenges faced by nurses and provide useful information for the development of appropriate policies and interventions. In turn, these policies can improve the working environment of healthcare workers, provide better social support and resources, and devise more effective management strategies to alleviate symptoms of depression and anxiety. Therefore, based on previous studies, we evaluated whether PSS and self-efficacy are related to anxiety and depression among Chinese nurses. Furthermore, we explored whether PSS has moderating effects on the associations between self-efficacy and anxiety and depression.

## 2. Materials and methods

We randomly selected 1,200 nurses from five tertiary level hospitals in Liaoning Province (240 nurses per hospital). Due to the scarcity of male nurses in China, this study focused on female nurses (14). The investigators obtained pre-survey support and cooperation from both hospitals and respondents. Respondents provided written informed consent before the questionnaire survey was administered. To ensure adequate validity of the responses, the questionnaires were collected face-to-face, and the investigators performed preliminary verification of both the number and content of the questionnaires. Nurses aged at least 18 years who had worked for at least a year were included in the study. Conversely, interns and nurses who had worked for less than a year were excluded. We received 1,010 completed questionnaires, correlating with a participation rate of 84.2%.

### 2.1. Ethics statement

The studies involving human participants were reviewed and approved by Ethical Committee of Dalian Medical University. The patients/participants provided their written informed consent to participate in this study. Participants’ data remained confidential and anonymous to protect their privacy.

### 2.2. Sample size

We calculated the sample size using the average sampling formula: *N* = [Uασ/δ]^2^, where *α* = 0.05, *δ* = 0.1, and U*α* = 1.96. Based on the pre-survey standard deviation *σ* = 1.52, we calculated the minimum sample size as 887, taking into account the potential sampling error and a missing rate of 20%. Therefore, we distributed and collected 1,200 questionnaires and obtained 1,010 valid responses, thereby meeting the minimum sample requirement.

### 2.3. Demographic variables

This study evaluated five sociological variables: age, province, marital status, number of years of work experience, and health status. Age was categorized as <32 and ≥ 32 years. Province was categorized as Liaoning Province and other provinces. Marital status was categorized as married or single/widowed/divorced. The number of years of work experience were categorized as <10 and ≥ 10. Health status was categorized as poor or good.

### 2.4. Measures

The General Self-Efficacy Scale (GSES) is used to assess nurses’ self-efficacy (30). It has demonstrated good internal consistency in the present study (Cronbach’s *α* = 0.90). This scale consists of 10 items, each with four options (1 = not at all true to 4 = exactly true), with total scores ranging from 10 to 40 and higher scores indicating greater self-efficacy.

The Multidimensional Scale of Perceived Social Support (MSPSS) is used to assess nurses’ PSS (31). In the current study, the scale had a Cronbach’s *α* of 0.96. It consists of 12 items, each scored on a 7-point Likert scale, with scores ranging from 12 to 84. The scale assesses nurses’ overall social support, with higher composite scores indicating better social support.

The 14-item Hospital Anxiety and Depression Scale (HADS) is used to assess anxiety and depressive symptoms in nurses (32). It consists of a 7-item anxiety subscale and a 7-item depression subscale. Participants are instructed to select a score from 0 (not at all) to 3 (very much), with subscales ranging from 0 to 21 and higher scores indicating greater anxiety or depression. In our study, HADS had acceptable internal consistency (Cronbach’s *α* = 0.85).

### 2.5. Statistical analyzes

Data were collected and analyzed using SPSS. All variables were compared between groups, with *p* < 0.05 considered statistically significant. The associations between anxiety and depression scores and categorical variables were evaluated using independent samples *t*-tests, whereas the associations between age, self-efficacy, PSS, anxiety, and depression were determined using Pearson correlation analysis. Hierarchical multiple regression analysis was used to examine factors influencing anxiety and depression, whereas the moderating effects of PSS on self-efficacy and mental health were examined using bias-corrected nonparametric percentile bootstrapping in the PROCESS macro (version 3.0 by Andrew F. Hayes) for SPSS. Hierarchical multiple regression was performed in three steps, with the five sociodemographic variables as control variables, PSS and self-efficacy, and self-efficacy and PSS interaction terms being entered sequentially into the regression model. If the interaction term was significant, our hypothesis of a moderating effect of PSS was tested and visualized using simple slope analysis. To account for multicollinearity, the variance inflation factor (VIF) was used, with VIF < 10 indicating no significant multicollinearity.

## 3. Results

In this study, the mean depression and anxiety scores among nurses were 8.74 ± 3.50 and 6.18 ± 3.26, respectively. The univariate analysis between demographic characteristics and psychological well-being is presented in Table 1. The mean age of the 1,010 participants was 33.25 ± 7.28 years, with 47.33% of nurses aged >32 years old. The majority of nurses (75.94%) originated from the Liaoning province, whereas 24.06% originated from other provinces. Only 30.99% of the nurses were married, and less than half of the nurses had an experience of more than 10 years (46.04%). Furthermore, 67.33% of the nurses had a poor health status. Among the five demographic variables, only health status was related to anxiety (*p* < 0.01), and nurses with a poor health status had higher anxiety scores. Marital and health statuses were related to depression. Married nurses had low depression scores (*p* < 0.05), whereas nurses with a poor health status had high depression scores (*p* < 0.01).

**Table 1:** Demographic characteristics of nurses and univariate analysis of factors associated with anxiety and depression levels.

Variable		*N* (%)	Mean ± SD	
			Depression	Anxiety
Age (years)	< 32	532 (52.67)	8.67 ± 3.62	6.20 ± 3.23
	≥ 32	478 (47.33)	8.82 ± 3.37	6.16 ± 3.31
*p*-value			0.49	0.85
Province	Liaoning province	767 (75.94)	8.63 ± 3.49	6.14 ± 3.26
	Other provinces	243 (24.06)	9.07 ± 3.53	6.28 ± 3.30
*p*-value			0.09	0.58
Marital status	Married	313 (30.99)	8.38 ± 3.64	6.27 ± 3.24
	Single/widowed/divorced	697 (69.01)	8.90 ± 3.43	6.14 ± 3.28
*p*-value			0.03	0.57
Years of experience	< 10	545 (53.96)	8.70 ± 3.62	6.31 ± 3.25
	≥ 10	465 (46.04)	8.78 ± 3.36	6.03 ± 3.28
*p*-value			0.72	0.17
Health status	Poor	680 (67.33)	9.29 ± 3.24	6.80 ± 3.23
	Good	330 (32.67)	7.60 ± 3.74	4.9 ± 2.96
*p*-value			< 0.01	< 0.01

### 3.1. Correlations between continuous variables

Table 2 displays the correlations between the continuous variables. The mean general self-efficacy and PSS scores were 27.56 ± 4.15 and 67.94 ± 11.37, respectively. A significant negative correlation was observed between general self-efficacy and anxiety and depression (*r* = −0.242 and *r* = −0.250, respectively; *p* < 0.05). Additionally, PSS demonstrated significant negative correlations with anxiety and depression (*r* = −0.304 and *r* = −0.307, respectively; *p* < 0.05).

**Table 2:** Pearson’s correlation coefficient of age, self-efficacy, and PSS with depression and anxiety.

	Mean ± SD	1	2	3	4	5
Age (years)	33.25 ± 7.28	1				
Self-efficacy	27.56 ± 4.15	−0.013	1			
PSS	67.94 ± 11.37	0.023	0.312*	1		
Depression	8.74 ± 3.50	0.065*	−0.250*	−0.307*	1	
Anxiety	6.18 ± 3.26	0.008	−0.242*	−0.304*	0.531*	1

*p < 0.05.

### 3.2. Associations of sociodemographic characteristics, self-efficacy, and PSS with the severity of psychological symptoms using hierarchical multiple regression analysis

**Figure 1:**
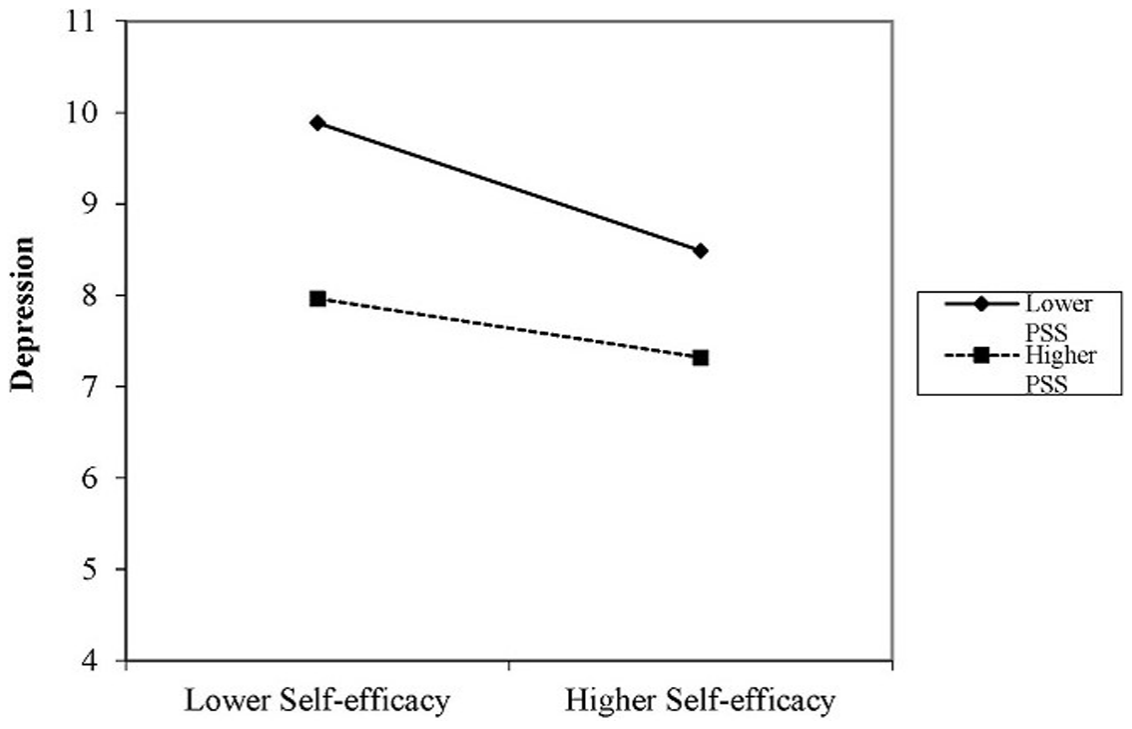
Simple slope plot of the interaction between self-efficacy and PSS on depression/anxiety.

As shown in Table 3, age, province, marital status, number of years of experience, and health status significantly explained the level of depression in the first step of regression analysis (adjusted *R*^2^ = 0.055, *p* < 0.01). Following the exclusion of the effects of the control variables in the second step, self-efficacy and PSS exhibited significant negative associations with depression (*β* = −0.128, *p* < 0.01 and *β* = −0.071, *p* < 0.01, respectively). In the third step, depression was significantly positively correlated with the interaction term (*β* = 0.004, *p* < 0.05; Table 3). Simple slope analysis indicated that increasing PSS was associated with a reducing strength of association between self-efficacy and depression. In other words, self-efficacy exhibited differential effects on depression at low PSS (1 SD below the mean, *β* = −0.169, *p* < 0.01) and high PSS (1 SD above the mean, *β* = −0.077, *p* < 0.01). These interactions are illustrated in Figure 1.

**Table 3:** Relationship between sociodemographic characteristics, self-efficacy, PSS, and depression.

	Step 1	Step 2	Step 3
Step 1			
Age	0.032	−0.143	−0.136
Province	0.637*	0.523*	0.548*
Marital status	0.568*	0.732**	0.742**
Years of experience	−0.153	−0.012	−0.001
Health Status	−1.670**	−1.093**	−1.104**
Step 2			
Self-efficacy		−0.128**	−0.123**
PSS		−0.071**	−0.068**
Step 3			
Interaction			0.004*
F	9.379**	16.90**	16.45**
Adjusted R^2^	0.055	0.146	0.149
△R^2^	0.060	0.092	0.005

*p < 0.05, **p < 0.01.

**Figure 2:**
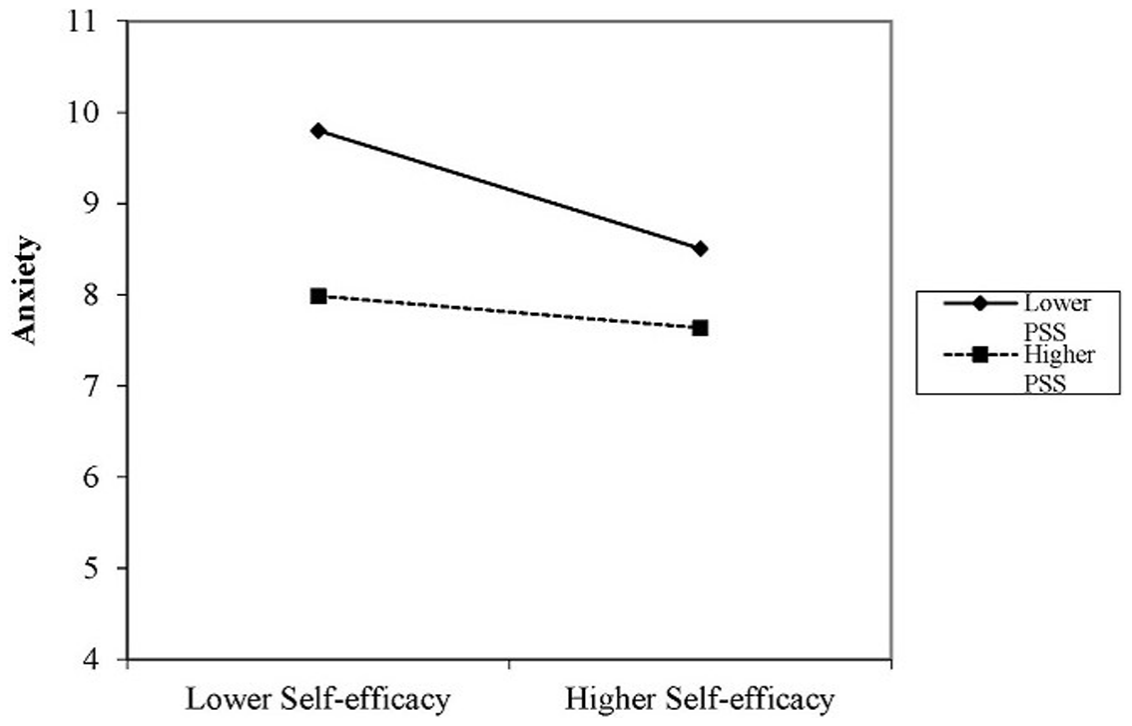
Simple slope plot of the interaction between self-efficacy and PSS on depression/anxiety.

Table 4 demonstrates that the control variables significantly explained the associations in the first step of regression analysis (adjusted *R*^2^ = 0.08, *p* < 0.01). In the second step, after controlling for other variables, self-efficacy and PSS were significantly negatively associated with anxiety (*β* = −0.106, *p* < 0.01 and *β* = −0.062, *p* < 0.01, respectively). The self-efficacy × PSS interaction term was significantly positively associated with anxiety in the third step (*β* = 0.005, *p* < 0.01). Additionally, simple slope analysis showed that the strength of the relationship between self-efficacy and anxiety decreased with increasing PSS. In particular, the impact of self-efficacy on anxiety varied between low PSS (1 SD below the mean, *β* = −0.155, *p* < 0.01) and high PSS (1 SD above the mean, *β* = −0.044, *p* < 0.01). These interactions are displayed in Figure 2.

**Table 4:** Relationship between sociodemographic characteristics, self-efficacy, PSS, and anxiety.

	Step 1	Step 2	Step 3
Step 1			
Age	0.391	0.239	0.248
Province	0.073	−0.027	0.003
Marital status	−0.190	−0.0479	−0.036
Years of experience	−0.645*	−0.524	−0.512
Health Status	−1.941**	−1.447**	−1.461**
Step 2			
Self-efficacy		−0.106**	−0.100**
PSS		−0.062**	−0.059**
Step 3			
Interaction			0.005**
F	9.35**	15.77**	15.23**
Adjusted R^2^	0.076	0.152	0.160
△R^2^	0.081	0.078	0.008

*p < 0.05, **p < 0.01.

## 4. Discussion

This study examined the levels of anxiety and depression among Chinese nurses. Our results indicated that the anxiety and depression scores among participants were 6.18 ± 3.26 and 8.74 ± 3.50, respectively, which are higher than those reported previously (33), suggesting that Chinese nurses have serious mental health problems and need effective interventions. We identified an inverse correlation between self-efficacy, PSS, and psychological disorders among nurses. Choi et al. demonstrated that self-efficacy reduced the risk of psychological disorders in nurses (34). Chen et al. (8) confirmed a negative association between PSS and psychological disorders, with individuals with high PSS demonstrating lower risks of anxiety and depression. In addition, PSS plays a moderating role between nurses’ self-efficacy and psychological well-being, which is consistent with the results of Shahzad et al. (35). Mental health problems exhibit significant gender differences, with women reporting more severe anxiety and depressive symptoms (36). Therefore, it is important to explore strategies that can protect the mental health of female nurses, given their high levels of occupational stress.

First, in the present study, we identified a negative correlation between the self-efficacy of the nurses and their anxiety and depression. Similarly, Xiong et al. demonstrated that self-efficacy of nurses working in public hospitals had a positive effect on their psychological status. Furthermore, improving the self-efficacy of nurses enhanced their ability to cope with new infectious diseases and reduced their depression and anxiety symptoms (37), which is consistent with our study. Self-efficacy is an intrinsic personality trait that is associated with a high confidence level. Individuals with low self-efficacy do not have the motivation or resources to act effectively in the face of challenges. Therefore, the self-efficacy of nurses is an important predictor of their emotional responses, choice behaviors, and thought patterns (38). Nurses with low levels of self-efficacy are unable to adapt to the complex and stressful work environment of hospitals, which can contribute to anxiety and depression (39). Bandura’s social cognitive theory posits that high levels of self-efficacy play an essential part in managing anxiety, depression, and other emotional states. This may be because nurses with high self-efficacy believe that they can control stressors and are less likely to experience negative emotions, such as anxiety and depression; these nurses have better nursing skills and can handle challenges more confidently and calmly (16). In contrast, nurses with lower self-efficacy may overestimate their challenges and worry excessively about negative consequences. Therefore, it is essential to strengthen nurses’ self-efficacy to improve their psychological well-being.

Our study also found that nurses’ PSS was negatively associated with anxiety and depression. In a study of the mental health of healthcare workers in Jordan, PSS was found to be an essential coping mechanism that reduced nurses’ psychological distress and promoted positive emotions (40). The buffering model of social support suggests that social support can alleviate negative emotions and improve mental health. Social support has multiple positive effects, which may be related to its role in enhancing the psychological wellbeing, reducing adverse emotions such as anxiety and depression, or cushioning the negative impacts of an event on an individual (41). It is important for nurses to understand the concept of social support because underestimating the effects of social support can lead to a loss of its impact on an individual, regardless of the level of physical and emotional support provided (42). The role of PSS is particularly important given the complexity and demanding nature of the hospital work environment. A good PSS network can improve access to positive psychological resources that can counteract the negative emotions of anxiety and depression. In addition, PSS can offer nurses problem-solving tactics, minimize the significance of issues, and alleviate any related adverse impacts (43).

Importantly, we found a moderating effect of PSS on the association between self-efficacy and mental health. The simple slope plots showed that increasing PSS was negatively correlated with the strength of association between self-efficacy and anxiety and depression. In conclusion, the effects of nurses’ self-efficacy on their psychological symptoms varied with the level of PSS, suggesting that greater PSS can promote the mental health of nurses with lower self-efficacy and reduce the occurrence of anxiety and depression. However, with increasing PSS, the strength of associations between self-efficacy and anxiety and depression weakened, possibly due to the effects of other positive psychological resources, such as optimism and self-esteem, when accessing PSS (44). Therefore, PSS, a modifiable moderator among nurses, should be improved by effective interventions to enhance nurses’ ability to cope with mental health problems.

Medical professionals play a vital role in maintaining and improving the health of the population. They are often exposed to highly stressful work environments and are routinely confronted with illness, patient suffering, and life-threatening challenges (18). Therefore, it is crucial to evaluate the social support, self-efficacy, and depression and anxiety symptoms among healthcare workers to enhance their job satisfaction, reduce job stress, and boost their job effectiveness. This study examined the relationship between PSS, self-efficacy, and psychological disorders among Chinese nurses. We found significant negative correlations of self-efficacy and PSS with psychological disorders. Furthermore, PSS moderated the association between self-efficacy and psychological disorders. An increase in PSS was associated with a decreasing strength of association between self-efficacy and psychological disorders, indicating that the development of psychological disorders can be prevented among nurses with lower self-efficacy by increasing the PSS. In contrast to previous studies of the prevalence and influencing factors of mental illness among Chinese nurses, the present study comprehensively examined the effects of PSS as an external resource and self-efficacy as an internal resource on nurses’ mental health. Our results suggest feasible strategies to prevent mental illness among nurses by urging hospital administrators to provide better training and social support, thereby improving nurses’ self-efficacy and enhancing their adaptability and stress resistance. These measures can improve healthcare workers’ career development and quality of life, ultimately improving the level and quality of healthcare provided. Our findings have practical implications for enhancing nurses’ mental health and emphasize the importance of self-efficacy and PSS. Governments should improve the protection system for nurses, establish independent academic organizations, and strengthen nurses’ sense of solidarity. Hospitals should alleviate the negative emotions of nurses, such as anxiety and depression, whereas nurse managers should pay attention to nurses’ feedback and support nurses. Nurses with low self-efficacy should recognize their deficiencies and improve their work competencies by enhancing their confidence (45). Hospitals should provide systematic organizational support, enhance opportunities for support at all levels within the organization, provide clinical clubs for nurses, and organize learning and training sessions. Furthermore, nurses should develop an extensive support network outside of work, where they can actively participate in social activities and share their experiences with their spouses, to gain their spouses’ understanding and support (46).

The present study had several limitations. First, the causal relationship between nurses’ self-efficacy, PSS, anxiety, and depression could not be inferred from this cross-sectional study. Therefore, longitudinal studies are needed to verify their relationship. Second, this study included female nurses only. Therefore, future studies should include both male and female nurses. Finally, we only selected nurses from the Liaoning Province, which may lead to limited generalizability of our results.

## 5. Conclusion

Anxiety and depression are prevalent among Chinese nurses. We found that self-efficacy and PSS are negatively associated with anxiety and depression. Interventions to improve self-efficacy and PSS can alleviate the effects of anxiety and depression on nurses.

## Data availability statement

The original contributions presented in the study are included in the article/supplementary material, further inquiries can be directed to the corresponding author.

## Ethics statement

The studies involving human participants were reviewed and approved by Ethical Committee of Dalian Medical University. The patients/participants provided their written informed consent to participate in this study.

## Author contributions

JL wrote the first draft of the manuscript. XD, YY, and BW edited the paper. YZ, HJ, and XC revised the manuscript. MS, YD, and YP analyzed the research data. YC and YY helped with visualization and investigation. LZ helped with project administration, resources, and writing. All authors contributed to the article and approved the submitted version.

## Conflict of interest

The authors declare that the research was conducted in the absence of any commercial or financial relationships that could be construed as a potential conflict of interest.

## Publisher’s note

All claims expressed in this article are solely those of the authors and do not necessarily represent those of their affiliated organizations, or those of the publisher, the editors and the reviewers. Any product that may be evaluated in this article, or claim that may be made by its manufacturer, is not guaranteed or endorsed by the publisher.

## Abbreviations

GSES: General Self-Efficacy Scale
HADS: Hospital Anxiety and Depression Scale
MSPSS: Multidimensional Scale of Perceived Social Support
PSS: Perceived social support
SW: Shift work
VIF: Variance inflation factor
